# Oncogenic circular RNA circ_0007534 contributes to paclitaxel resistance in endometrial cancer by sponging miR-625 and promoting ZEB2 expression

**DOI:** 10.3389/fonc.2022.985470

**Published:** 2022-08-04

**Authors:** Hanjie Yi, Yongqing Han, Shanfeng Li

**Affiliations:** ^1^ Department of Oncology, The Second Affiliated Hospital of Nanchang University, Nanchang, China; ^2^ Department of Oncology, ShangRao People’s Hospital, Shangrao, China; ^3^ Department of Nosocomial Infection Management, The Second Affiliated Hospital of Nanchang University, Nanchang, China

**Keywords:** circ_0007534, miR-625, ZEB2, EMT, paclitaxel resistance, endometrial cancer

## Abstract

Circular RNAs (circRNAs) and epithelial to mesenchymal transition (EMT) have been implicated in the development of human cancer and paclitaxel resistance. CircRNA circ_0007534 has been described as a key oncogenic circular RNA that is upregulated in a variety of cancer tissues. However, whether circ_0007534 causes EMT and paclitaxel resistance in endometrial cancer is still unknown. In this work, we revealed that circ_0007534 levels were significantly higher in endometrial cancer tissues, and that high circ_0007534 expression was associated with poor differentiation, advanced tumor stage, cancer invasion, cancer metastasis, and poor prognosis in endometrial cancer patients. Overexpression of circ_0007534 boosted endometrial cancer cell proliferation, invasion, EMT, and paclitaxel resistance. Knockdown of circ_0007534 restored paclitaxel sensitivity and reversed EMT in endometrial cancer cells. We also showed that circ_0007534 enhanced endometrial cancer aggressiveness, progression, and paclitaxel resistance by sponging microRNA-625 (miR-625) and subsequently increasing the expression of the miR-625 target gene *ZEB2*. Our cell functional studies demonstrated that inhibiting miR-625 or increasing ZEB2 mimicked the effects of circ_0007534 overexpression. Consequently, our data show that circ_0007534 plays a crucial role in EMT and paclitaxel resistance through miR-625/ZEB2 signaling. Targeting the circ_0007534/miR-625/ZEB2 pathway might be an effective strategy for overcoming paclitaxel resistance in endometrial cancer.

## Introduction

Endometrial cancer is the most prevalent gynecological malignancy and the second leading cause of cancer-related mortality in women worldwide (1). Although the majority of endometrial cancer patients are identified with early-stage illness, which is often treatable with surgery, sometimes in conjunction with adjuvant treatment (2), the prognosis for metastatic and advanced illness is dismal, with a 5-year survival rate of 15 to 17% (3). Determining the molecular basis of endometrial cancer formation and progression will thus contribute to the development of diagnostic and therapeutic strategies to enhance survival.

EMT is a key process that has been linked to enhanced cervical cancer invasion, metastasis, and chemoresistance and is characterized by a loss of epithelial markers such as E-cadherin and an upregulation of mesenchymal markers such as vimentin (4). Furthermore, several transcription factors, including Twist1, ZEB1, ZEB2, and Snail/Slug families, are involved in the EMT process (4). ZEB2 is important in EMT-related processes such as cancer growth, drug resistance, cancer stem cells, apoptosis, survival, cell cycle arrest, cancer recurrence, and metastasis (5). ZEB2 can trigger EMT in endometrial cancer by raising vimentin levels while decreasing E-cadherin expression (6). Moreover, the relationship between EZB2 overexpression and chemoresistance is increasingly established (7, 8). The abrogation of ZEB2 increased paclitaxel sensitivity of lung cancer and hepatocellular carcinoma (9, 10). To the best of our knowledge, there is no detailed report on the relationship between ZEB2 and paclitaxel resistance in endometrial cancer.

Non-coding RNA molecules, such as microRNAs (miRNAs) and circular RNAs (circRNAs), have been shown to modulate gene expression and cancer cell susceptibility to paclitaxel (11, 12). MiR-212 was expressed at low levels in Paclitaxel-resistant hepatocellular carcinoma cells (10). By binding to the 3´-untranslated region (3´-UTR) of target *ZEB2* mRNA, miR-212 restored paclitaxel sensitivity in hepatocellular carcinoma cells (10). MiR-625 expression was significantly lower in endometrial cancer tissues than in matching normal samples, and circ_002577 enhanced endometrial cancer development by functioning as a sponge for miR-625, elevating IGF1R expression, and activating the PI3K/Akt pathway (13). MiR-625 has been found to inhibit cancer cell proliferation, migration, invasion, and EMT in lung cancer and laryngeal squamous cell carcinoma (14, 15). However, the regulatory association between miR-625 and Paclitaxel resistance in endometrial cancer and the mechanisms underlying miR-625-mediated EMT and Paclitaxel resistance are completely unknown.

Moreover, circRNAs are a type of circular RNA that is covalently closed and more stable than linear RNAs (16–18). CircRNA expression is tissue- and cell-specific, and it regulates a variety of physiological and pathological activities, such as EMT, metastasis, and chemoresistance (16–18). In previous studies, the expression of hsa_circ_0007534 was observed to be significantly higher in colon, breast, cervical and pancreatic cancers than in normal tissues (19–22). It was reported that circ_0007534 increases cell proliferation and invasion in pancreatic cancer (22). The importance of circ_0007534 and its downstream pathway in endometrial cancer, however, is yet uncertain.

In this work, we report an essential function of circ_0007534 in endometrial cancer, in which circ_0007534 acts as a sponge for miR-625 to increase ZEB2 levels, hence promoting EMT and Paclitaxel resistance in endometrial cancer. Consequently, the present study indicated that the circ_0007534/miR-625/ZEB2 pathway might be a significant therapeutic target for overcoming Paclitaxel resistance in endometrial cancer.

## Materials and methods

### Human tissue specimens

Endometrial cancer samples and adjacent normal samples were acquired from patients who received surgery in the Second Affiliated Hospital of Nanchang University. None of the patients were treated with preoperative chemotherapy or radiotherapy. This study was approved by the Research Ethics Committee of Second Affiliated Hospital of Nanchang University, and all patients provided written informed consent. Tissue samples were immediately snap-frozen in liquid nitrogen and stored at -80°C.

### Cell Lines and cell culture

Human endometrial cancer cell lines (Ishikawa, RL95 and HEC-1) and a human normal endometrial fibroblast cell line HESC were purchased from the American Type Culture Collection (ATCC, Manassas, VA). Dulbecco’s modified Eagle’s medium/nutrient mixture F-12 (DMEM/F12, Sigma-Aldrich, St. Louis, MO, USA) supplemented with 10% fetal bovine serum (FBS, Thermo Fisher Scientific, Waltham, MA, USA) was used to culture Ishikawa, RL95, HEC-1, and HESC cells. All cell lines were maintained in a humidified 5% CO_2_ incubator at 37°C.

### RNA preparation and real-time PCR

TRIzol reagent (Thermo Fisher Scientific) was utilized to extract total RNA from tissue samples or cells. Cytoplasmic and nuclear RNA was separated using a PARIS kit (Thermo Fisher Scientific). For RNase R treatment, 2 μg of total RNA was incubated with or without 3 U/μg RNase R (Geneseed Biotech, Guangzhou, China) for 10 minutes at 37°C. For the measurement of mRNA and circRNAs, cDNA was synthesized using a PrimeScript reagent kit (TaKaRa, Beijing, China).

Quantitative real-time PCR was performed using SYBR Green (TaKaRa) with the following primers: hsa_circ_0007534 forward: 5′-GTGACGGAAATCCAATTGCACC-3′ and hsa_circ_0007534 reverse: 5′-ATGGAATTGCTGGCGAGTTG-3′; *DDX42* forward: 5′- ACTAAGCGAGGATTTGGCTTTG-3′ and *DDX42* reverse: 5′- GGACTGCTGTGGGAGTTTGG-3′; *E-cadherin* (*CDH1*) forward: 5′-CGAGAGCTACACGTTCACGG-3′ and *E-cadherin* reverse: 5′-GGGTGTCGAGGGAAAAATAGG-3′; *Vimentin* (*VIM*) forward: 5′-GACGCCATCAACACCGAGTT-3′ and *Vimentin* reverse: 5′-CTTTGTCGTTGGTTAGCTGGT-3′; *Twist1* forward: 5′-GTCCGCAGTCTTACGAGGAG-3′ and *Twist1* reverse: 5′-GCTTGAGGGTCTGAATCTTGCT-3′; *MMP2* forward: 5′-TACAGGATCATTGGCTACACACC-3′ and *MMP2* reverse: 5′-GGTCACATCGCTCCAGACT-3′; *ZEB2* forward: 5′-CAAGAGGCGCAAACAAGCC-3′ and *ZEB2* reverse: 5′-GGTTGGCAATACCGTCATCC-3′; *GAPDH* forward: 5′-AATCCCATCACCATCTTC-3′ and *GAPDH* reverse: 5′-AGGCTGTTGTCATACTTC-3′. *GAPDH* was used as the normalization control.

The NCode SYBR GreenER miRNA qRT-PCR analysis kit (Thermo Fisher Scientific, Waltham, MA) was used to evaluate miR-625 expression. The forward primer was as follows: 5′-AGGGGGAAAGTTCTATAGTCC-3′. The universal reverse primer was provided in the NCode SYBR GreenER miRNA qRT-PCR analysis kit. The endogenous control was U6 (U6 forward: 5′-GCTTCGGCAGCACATATACTAAAAT-3′ and U6 reverse: 5′-CGCTTCACGAATTTGCGTGTCAT-3′).

### Plasmid construction

Human full-length circ_0007534 was amplified and inserted into pLCDH-ciR (Geenseed Biotech), generating the circ_0007534 overexpression vector (circ_0007534 vector). For the knockdown experiments, circ_0007534 siRNA (5′-GATCATTCAGAGCTATTTTGA-3′) or scramble control sequence (5’-TTCTCCGAACGTGTCACGT-3’) was synthesized and cloned into the pLent-U6-GFP-Puro vector (Mailgene Biosciences, Beijing, China) to generate circ_0007534 shRNA vector or control shRNA, respectively.

To establish a stable circ_0007534-knockdown HEC-1 cell line or Ishikawa cells stably overexpressing circ_0007534, we cotransfected the above vectors or control vector with packaging plasmids (psPAX2 and pMD2. G, Geneseed Biotech) into HEK293 cells using Lipofectamine 3000 (Thermo Fisher Scientific). After 48 hours, the packaged lentiviruses were harvested. Endometrial cancer cells were infected with the virus and selected with 0.5 μg/ml puromycin (Sigma-Aldrich). The siRNA targeting ZEB2 and control siRNA were obtained from Ambion (Thermo Fisher Scientific). The ZEB2 overexpression vector and control vector were purchased from Origene (Rockville, MD, USA). Transient transfection was conducted using Lipofectamine 3000 (Thermo Fisher Scientific).

### Cell proliferation assay

Cell Counting Kit-8 (CCK-8, Dojindo, Japan) was used to examine the proliferation of endometrial cancer cells. In brief, 1000 cells were grown in 96-well plates and cultured for 96 hours. Then, CCK-8 solution (10 µl) was added to each well, and the plates were incubated at 37°C for 2 hours. At 450 nm, the absorbance was finally measured.

### Xenograft tumorigenesis

Animal experiments were approved by the Animal Care and Use Committee of the Second Affiliated Hospital of Nanchang University. Nude mice, aged 4-5 weeks, were purchased from Shanghai Slac Laboratory Animal (Shanghai, China). For the tumorigenesis assay, the nude mice were injected subcutaneously with 1 × 10^6^ HEC-1 cells with circ_0007534 knockdown or corresponding control cells, or with Ishikawa stably overexpressing circ_0007534 or corresponding control cells. The size of bearing tumors was measured, and tumor volume was calculated using the formula: Volume = 0.5 × length × width^2^. On day 21, the mice were sacrificed, and tumors were harvested.

### Invasion assay

The invasion assay experiments were performed in Transwell chambers (Corning, NY, USA) with 8-µm pore insets. In brief, the lower chamber was filled with DMEM/F12 medium containing 10% FBS. Subsequently, 5 × 10^4^ endometrial cancer cells in serum-free medium were plated into the upper inserts. After the cells were incubated for 24 hours, the upper side of the membrane was wiped off. After that, the invaded cells were stained with crystal violet (Sigma–Aldrich) for 30 minutes. ImageJ software was used for cell counting.

### Western blot

Cells were lysed on ice in the RIPA lysis buffer (Thermo Fisher Scientific). The total protein concentration was determined using a BCA assay kit (23250, Pierce, MA, USA). Equal amounts of protein were separated by 12% SDS-PAGE and transferred to PVDF membranes (GE Healthcare Life Sciences, Piscataway, NJ). After blocking, the membranes were treated with primary antibodies overnight at 4°C. The following antibodies were used: anti-E-cadherin (1:1000, ab40772, Abcam, CA, USA), anti-Vimentin (1:1000, #5741, Cell Signaling Technology, Danvers, MA, USA), anti-ZEB2 (1:1000, ab191364, Abcam), and anti-β-actin (1:5000, #4967, Cell Signaling Technology). The next day, the membranes were further incubated with secondary antibodies, and the signals were detected by ECL-enhanced chemiluminescence solution (Millipore, Billerica, MA, USA).

### Cell viability assay

Cell viability was tested using the CCK-8 assay. Endometrial cancer cells were plated into 96-well plates and then exposed to various dosages of Paclitaxel (Sigma–Aldrich) for 48 hours. Following treatment, the optical density values were detected at a wavelength of 450 nm.

### Caspase-3/7 analysis

Endometrial cancer cells were plated at 5000 cells per well in 96-well plates and treated the next day with Paclitaxel. A caspase-Glo 3/7 assay (Promega, Southampton, UK) was used to detect caspase-3/7 activity 48 hours after treatment.

### Dual-luciferase reporter assay

The sequences of wild-type (WT) circ_0007534, mutant (MUT) circ_0007534, WT *ZEB2* 3´-UTR, or MUT *ZEB2* 3´-UTR were cloned into the pGL3-basic vector (Promega, Madison, WI, USA) to generate reporter constructs. The above reporter vectors were co-transfected with miR-625 mimic, miR-625 inhibitor, or the respective controls (Ambion, Thermo Fisher Scientific) into endometrial cancer cells using Lipofectamine 3000 (Thermo Fisher Scientific). Cells were collected 48 hours after transfection, and luciferase activities were evaluated using the Dual-Luciferase Reporter Assay System (Promega, USA).

### Statistical analysis

Statistical analysis was performed with SPSS 20.0 (SPSS, Chicago, USA). The results are expressed as the mean ± standard deviation and were analyzed by Student’s *t*-test, Mann–Whitney U test, and one-way ANOVA. *P* < 0.05 was considered statistically significant.

## Results

### Increased circ_0007534 expression is correlated with the progression and poor prognosis of endometrial cancer

**Figure 1:**
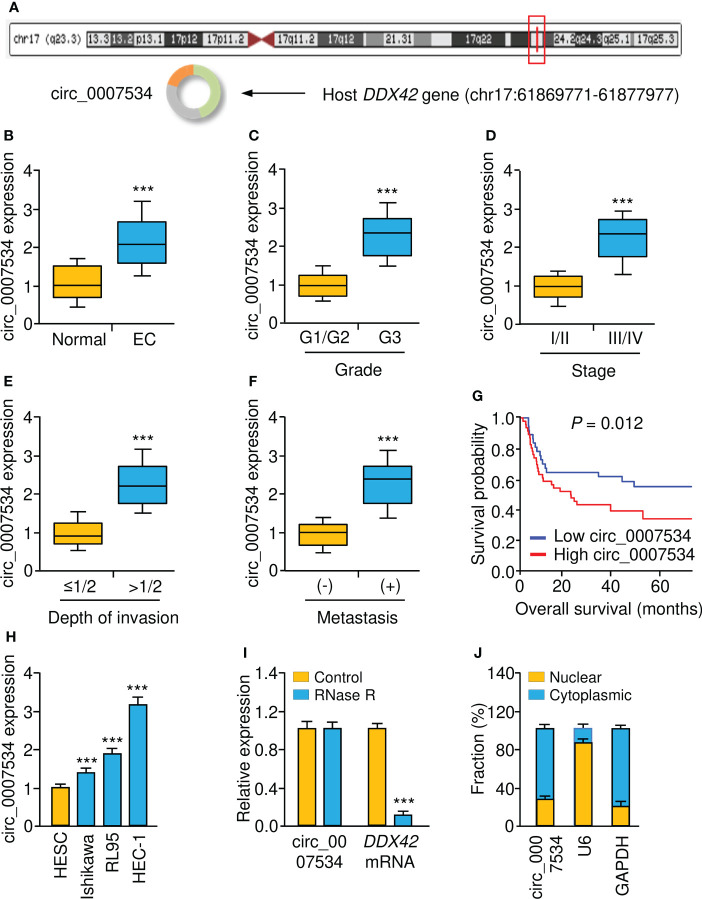
Upregulation of circ_0007534 is correlated with the progression and poor prognosis of endometrial cancer. **(A)** Diagram showed the formation of circ_0007534. **(B)** Real-time PCR detection of circ_0007534 expression in endometrial cancer and normal tissues. **(C-F)** Circ_0007534 expression in endometrial cancer (EC) patients with different tumor grades **(C)**, tumor stage **(D)**, depth of invasion **(E)**, and lymph node metastases **(F)** was examined using real-time PCRs. **(G)** The overall survival of endometrial cancer patients who had higher or lower circ_0007534 expression. **(H)** Circ_0007534 expression in endometrial cancer cells and normal cells. **(I)** Circ_0007534 was resistant to RNase R treatment in endometrial cancer cells. **(J)** The levels of circ_0007534 in the cytoplasm and nucleus of HEC-1 cells were examined using real-time PCR analysis. ***P < 0.001.

According to bioinformatics analysis using the CircInteractome, circRNADB, and circBase databases, we discovered circ_0007534 on chromosome 17, consisting of 18 exons (exons 2-19) from the *DDX42* gene (**Figure 1A**). We examined the levels of circ_0007534 in endometrial cancer tissues and corresponding normal tissues. Our real-time PCR results revealed that circ_0007534 levels in endometrial cancer tissues were significantly greater than those in normal tissues (**Figure 1B**). In particular, high circ_0007534 expression was detected in patients with poor tumor differentiation, late pathological stage, deeper invasion, and cancer metastasis (**Figures 1C-F**). According to the median circ_0007534 expression level, we classified endometrial cancer tissues into two groups: high expression and low expression. Patients with high circ_0007534 expression had a considerably shorter survival time, according to a Kaplan–Meier analysis (**Figure 1G**).

The expression level of circ_0007534 was then determined in several endometrial cancer cell lines as well as the normal cell line HESC. Circ_0007534 was also overexpressed in endometrial cancer cells relative to HESC cells (**Figure 1H**). After that, we found that after incubating the RNA with RNase R, the expression level of circ_0007534 remained unchanged; however, the expression level of *DDX42* was drastically reduced in endometrial cancer cells (**Figure 1I**). These results confirmed that circ_0007534 was RNase R resistant. Real-time PCR assays showed that circ_0007534 was mostly found in the cytoplasm of endometrial cancer cells (**Figure 1J**). All of these results indicated that upregulation of circ_0007534 is linked to endometrial cancer development and that circ_0007534 may have an oncogenic function in this disease.

### Circ_0007534 promotes the proliferation of endometrial cancer cells

**Figure 2:**
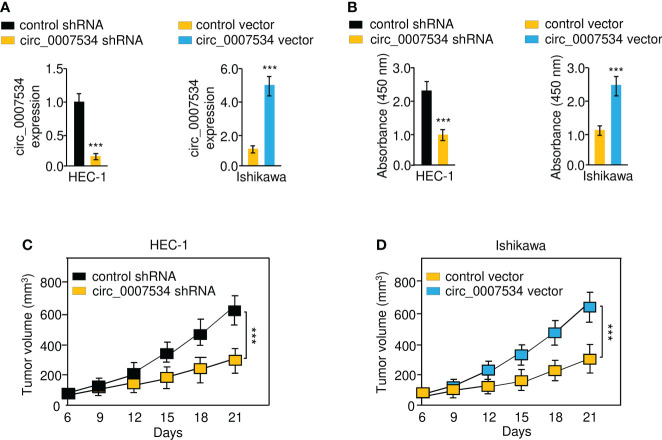
Circ_0007534 promotes the proliferation of endometrial cancer cells. **(A)** The expression of circ_0007534 in HEC-1 cells that were transfected with circ_0007534 shRNA or control shRNA (left) and in Ishikawa cells that were transfected with circ_0007534 vector or control vector (right). **(B)** The proliferation of the indicated cells was determined with the CCK-8 assay. **(C, D)** Effects of circ_0007534 knockdown or overexpression on xenograft HEC-1 **(C)** and Ishikawa **(D)** cell growth *in vivo*. ***P < 0.001.

First, we constructed stable HEC-1 cell lines with a knockdown of circ_0007534, Ishikawa cells overexpressing circ_0007534, and the respective control cell lines (**Figure 2A**). CCK-8 analysis revealed that after circ_0007534 knockdown, the proliferation of HEC-1 cells was significantly reduced (**Figure 2B**). However, overexpression of circ_0007534 significantly increased the growth of Ishikawa cells (**Figure 2B**). Then, we established xenograft tumor models in nude mice. Compared with the control group, the xenograft tumor growth rate was much slower in the circ_0007534 shRNA group (**Figure 2C**). Notably, the circ_0007534 vector group showed a higher growth rate than the control group (**Figure 2D**). These data suggested that the silencing of circ_0007534 inhibits the tumorigenicity of endometrial cancer cells.

### Circ_0007534 promotes invasion and EMT in endometrial cancer cells

**Figure 3:**
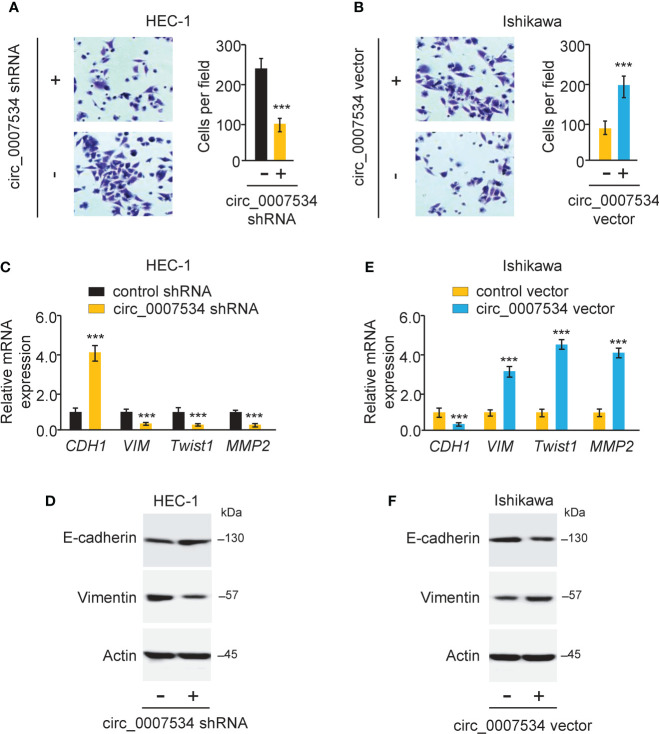
Circ_0007534 promotes invasion and EMT in endometrial cancer cells. **(A, B)** The invasion abilities of HEC-1 cells after silencing circ_0007534 **(A)** or Ishikawa cells after overexpression of circ_0007534 **(B)** were measured with an invasion assay. **(C, D)** The mRNA **(C)** and protein **(D)** expression of the indicated genes in HEC-1 cells was measured after silencing circ_0007534. **(E, F)** The mRNA **(E)** and protein **(F)** expression of the indicated genes in Ishikawa cells was examined after overexpression of circ_0007534. ***P < 0.001.

**Figure 4:**
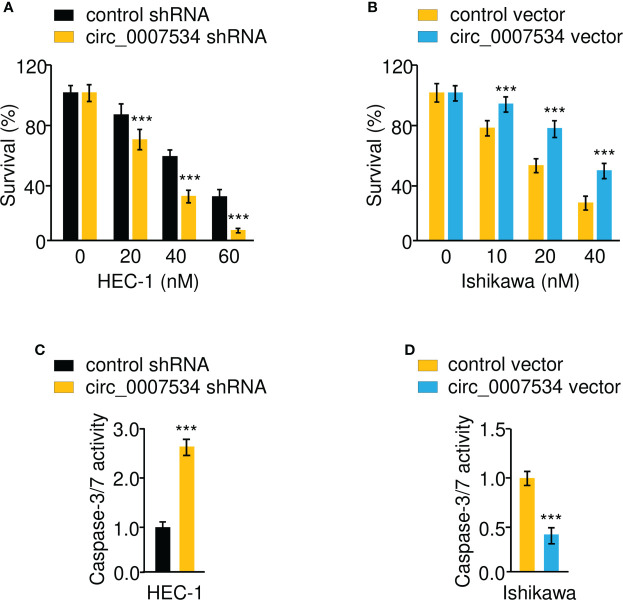
Circ_0007534 suppresses the sensitivity of endometrial cancer cells to paclitaxel. **(A, B)** The indicated HEC-1 **(A)** and Ishikawa **(B)** cells were treated with various concentrations of Paclitaxel for 24 hours, and cell viability was determined using a CCK-8 assay. **(C, D)** The activities of caspase-3 and caspase-7 in HEC-1 **(C)** and Ishikawa **(D)** cells were analyzed with Caspase-Glo 3/7 assays. ***P < 0.001.

We used a transwell invasion assay to investigate whether circ_0007534 knockdown or overexpression may affect the invasion of endometrial cancer cells. The invasion ability of HEC-1 cells was drastically reduced when circ_0007534 was knocked down (**Figure 3A**). After 24 hours of incubation, Ishikawa cells with circ_0007534 overexpression showed high invasive capabilities compared to control cells (**Figure 3B**). Detection of EMT-associated genes using real-time PCR and western blot analysis showed that the expression of E-cadherin was increased, whereas the levels of *Vimentin*, *Twist1*, and *MMP2* were suppressed in HEC-1 cells with circ_0007534 silencing (**Figures 3C, D**). However, Ishikawa cells overexpressing circ_0007534 had lower expression of E-cadherin and higher expression of *Vimentin*, *Twist1*, and *MMP2* than control cells (**Figures 3E, F**). Furthermore, circ_0007534 knockdown improved the susceptibility of HEC-1 cells to the chemotherapeutic agent Paclitaxel, whereas overexpression of circ_0007534 significantly enhanced Paclitaxel resistance in Ishikawa cells (**Figures 4A, B**). The contribution of circ_0007534 to cell apoptosis was tested using a caspase-Glo 3/7 assay. HEC-1 cells with circ_0007534 knockdown had significantly higher caspase-3/7 activities than the control cells (**Figure 4C**). However, overexpression of circ_0007534 in Ishikawa cells caused a significant decrease in cell apoptosis (**Figure 4D**). These results demonstrated that circ_0007534 facilitates EMT and suppresses the sensitivity of endometrial cancer cells to Paclitaxel.

### Circ_0007534 sponges MiR-625 in endometrial cancer cells

**Figure 5:**
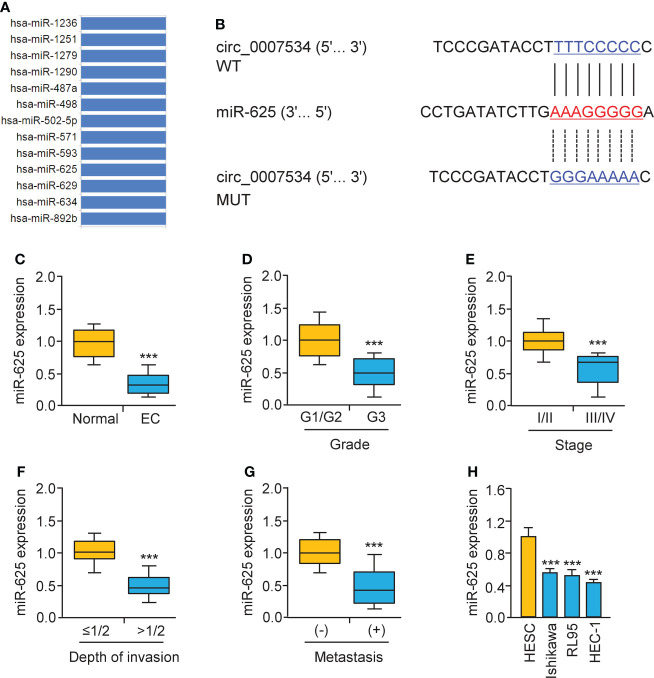
Low miR-625 levels are associated with the malignant progression of endometrial cancer. **(A)** The miRNAs that might bind to circ_0007534 were predicted using the CircInteractome database. **(B)** The luciferase reporter plasmids contained wild-type (WT) and mutated (MUT) miR-625 binding sites in the circ_0007534 sequence. **(C)** Real-time PCR detection of miR-625 expression in endometrial cancer and normal tissues. **(D-G)** MiR-625 expression in endometrial cancer patients with different tumor grades **(D)**, tumor stage **(E)**, depth of invasion **(F)**, and the presence (or absence) of lymph node metastasis **(G)** were explored using qRT-PCRs. **(H)** MiR-625 expression in endometrial cancer cells and normal cells. ***P < 0.001.

**Figure 6:**
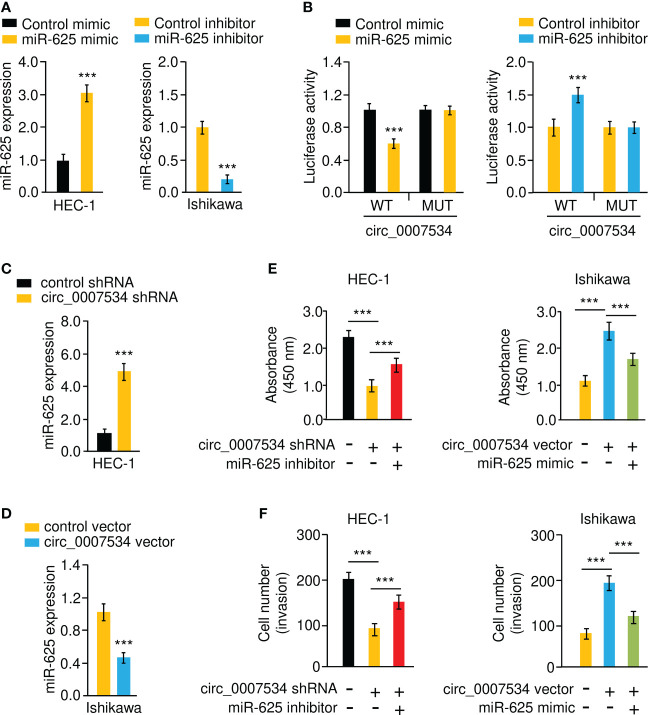
Circ_0007534 sponges miR-625 to promote endometrial cancer cell proliferation and invasion. **(A)** MiR-625 expression in endometrial cancer cells transfected with miR-625 mimic or inhibitor. **(B)** Luciferase reporter assay of endometrial cancer cells transfected with miR-625 mimic, miR-625 inhibitor, and luciferase reporter plasmids containing WT or MUT circ_0007534. **(C, D)** The expression of miR-625 in HEC-1 cells after knockdown of circ_0007534 **(C)**, or in Ishikawa cells after overexpression of circ_0007534 **(D)** was examined. **(E)** The proliferation of HEC-1 cells transfected with circ_0007534 shRNA, miR-625 inhibitor, or the respective controls (left). The proliferation of Ishikawa cells transfected with circ_0007534 vector, miR-625 mimic, or the respective controls (right). **(F)** The invasion of HEC-1 cells transfected with circ_0007534 shRNA, miR-625 inhibitor, or the respective controls (left). The invasion of Ishikawa cells transfected with circ_0007534 vector, miR-625 mimic, or the respective controls (right). ***P < 0.001.

Bioinformatic analysis with the CircInteractome database predicted that 13 miRNAs were potential circ_0007534 targets (**Figure 5A**). We noticed that the binding of miR-625 to circ_0007534 was also identified by the circRNADB database (**Figure 5A**). MiR-625 contained sequences complementary to circ_0007534 (**Figure 5B**). Real-time PCR assays confirmed that miR-625 levels were significantly downregulated in endometrial cancer samples compared with normal samples (**Figure 5C**). Moreover, the levels of miR-625 were decreased in endometrial cancer patients with poor tumor differentiation, late pathological stage, deeper invasion, and cancer metastasis (**Figures 5D-G**). Consistently, miR-625 was expressed at low levels in endometrial cancer cells compared with normal cells (**Figure 5H**). We created circ_0007534 luciferase reporter plasmids with miR-625 binding sites that were either wild-type (WT) or mutant (MUT) (**Figure 5B**). In HEC-1 cells co-transfected with miR-625 mimics and WT circ_0007534 (but not with MUT circ_0007534), the luciferase activity was significantly reduced (**Figures 6A, B**). Our results also suggested that in Ishikawa cells, the miR-625 inhibitor observably induced the luciferase activity of WT circ_0007534 (but not MUT circ_0007534) compared with the control inhibitor (**Figures 6A, B**). Furthermore, circ_0007534 overexpression reduced miR-625 expression, while circ_0007534 knockdown enhanced miR-625 expression in endometrial cancer cells (**Figures 6C, D**). Our cell function assays showed that the knockdown of miR-625 reversed the inhibitory effects of circ_0007534 silencing on cell proliferation and invasion (**Figures 6E, F**), and the miR-625 mimic reduced cell proliferation and invasion that was enhanced by circ_0007534 overexpression (**Figures 6E, F**). These findings revealed that circ_0007534 acts as a sponge for miR-625 to promote endometrial cancer cell proliferation and invasion.

### MiR-625 targets ZEB2 in endometrial cancer cells

**Figure 7:**
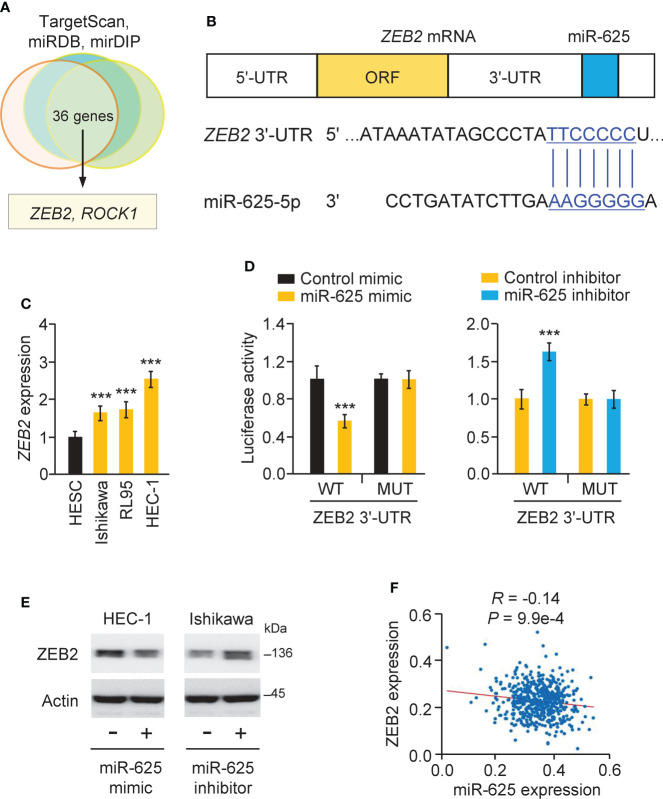
MiR-625 targets ZEB2 in endometrial cancer cells. **(A)** Venn analysis of predicted miR-625 target genes. **(B)** The luciferase reporter plasmids contained wild-type (WT) and mutated (MUT) miR-625 binding sites in the *ZEB2* 3´-UTR sequence. **(C)** *ZEB2* mRNA expression in endometrial cancer cells and normal cells. **(D)** Luciferase reporter assay of endometrial cancer cells transfected with miR-625 mimic, miR-625 inhibitor, and luciferase reporter plasmids containing WT or MUT *ZEB2* 3´-UTR. **(E)** Western blot analysis of ZEB2 and actin expression in endometrial cancer cells transfected as indicated. **(F)** The correlation between miR-625 and ZEB2 expression in TCGA endometrial cancer samples. ***P < 0.001.

The TargetScan, miRDB, and mirDIP databases were used to estimate the targets of miR-625, and the results suggested common target genes, including *ZEB2* and *ROC1* (**Figures 7A, B**). Since ZEB2 is a key EMT activator in endometrial cancer (6) and is possibly involved in chemoresistance in other tumor types (7, 8), we selected *ZEB2* and performed subsequent research. Using a real-time PCR assay, we proved that *ZEB2* levels were significantly higher in all endometrial cancer cells than in normal cells (**Figure 7C**). We used a luciferase reporter assay to confirm this prediction and found that the luciferase activity of the WT *ZEB2* 3´-UTR (but not the MUT *ZEB*2 3´-UTR) was reduced in HEC-1 cells co-transfected with the miR-625 mimic (**Figure 7D**). Transfection of the miR-625 inhibitor significantly increased the luciferase activity of the WT *ZEB2* 3´-UTR (but not the MUT *ZEB2* 3´-UTR) in Ishikawa cells (**Figure 7D**). Western blot assays showed that miR-625 overexpression downregulated ZEB2 expression, while the miR-625 inhibitor upregulated its expression (**Figure 7E**). Furthermore, endometrial cancer expression data were obtained from the TCGA study *via* the ENCORI database. The expression of miR-625 was negatively associated with the expression of ZEB2 in endometrial cancer tissues (**Figure 7F**). These data supported the inhibitory effects of miR-625 on ZEB2 expression.

### MiR-625 sensitizes the response to paclitaxel by regulating ZEB2 in endometrial cancer cells

**Figure 8:**
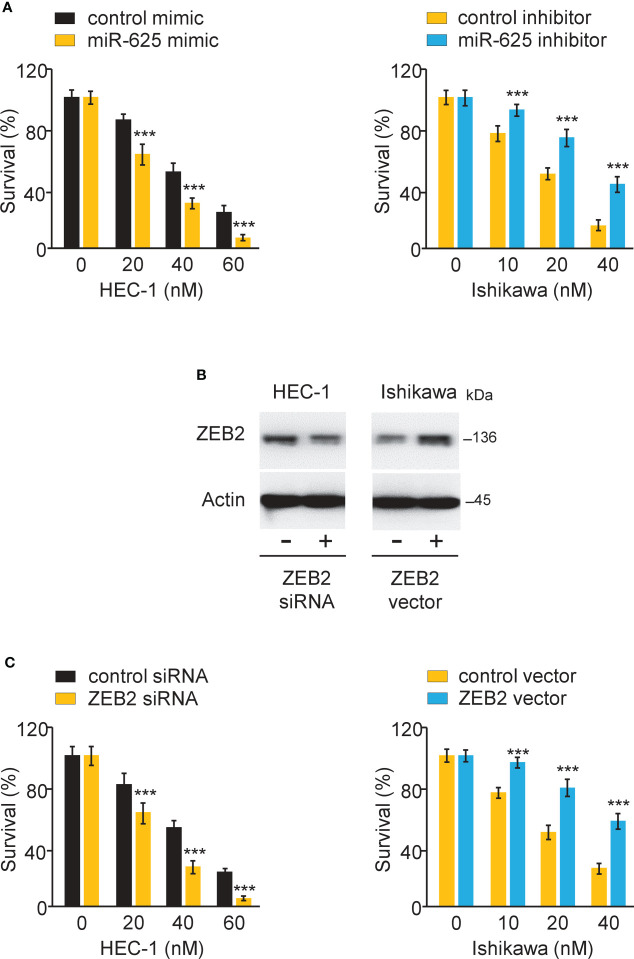
MiR-625 sensitizes the response to paclitaxel by regulating ZEB2 in endometrial cancer cells. **(A)** Endometrial cancer cells were transfected as indicated and treated with various concentrations of Paclitaxel, and cell viability was determined using a CCK-8 assay. **(B)** Western blot assay of ZEB2 and actin expression in endometrial cancer cells transfected as indicated. **(C)** Endometrial cancer cells were transfected as indicated and treated with various concentrations of Paclitaxel, and cell viability was determined using a CCK-8 assay. ***P < 0.001.

To investigate the effects of miR-625 or ZEB2 expression on Paclitaxel resistance, endometrial cancer cells were transfected with miR-625 mimic or inhibitor, as well as ZEB2 siRNA or control siRNA. CCK-8 assay was used to measure cell survival. Overexpression of miR-625 or siRNA-induced ZEB2 knockdown sensitized HEC-1 cells to Paclitaxel (**Figures 8A-C**). We also observed that inhibiting miR-625 expression or forced overexpression of ZEB2 significantly increased Paclitaxel resistance in Ishikawa cells (**Figures 8A-C**). The above results demonstrated that miR-625 regulates ZEB2 expression and reverses Paclitaxel resistance in endometrial cancer cells.

### Overexpression of ZEB2 induces EMT and increases endometrial cancer cell invasion

**Figure 9:**
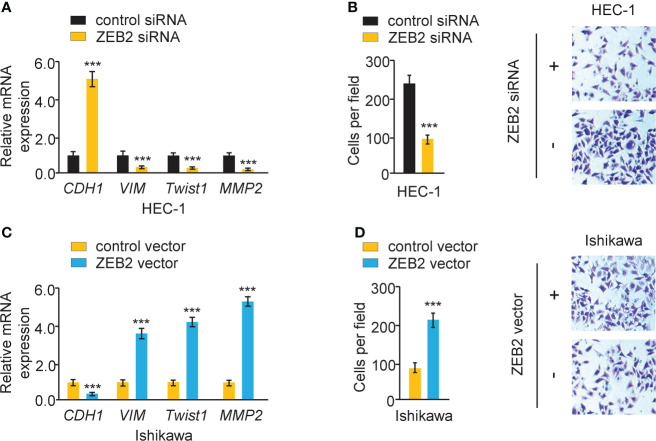
Overexpression of ZEB2 induces EMT and increases endometrial cancer cell invasion. **(A)** The mRNA expression of the indicated genes in HEC-1 cells after ZEB2 silencing. **(B)** The invasion abilities of HEC-1 cells after silencing ZEB2. **(C)** The mRNA expression of the indicated genes in Ishikawa cells after overexpression of ZEB2. **(D)** The invasion abilities of Ishikawa cells after overexpression of ZEB2. ***P < 0.001.

**Figure 10:**
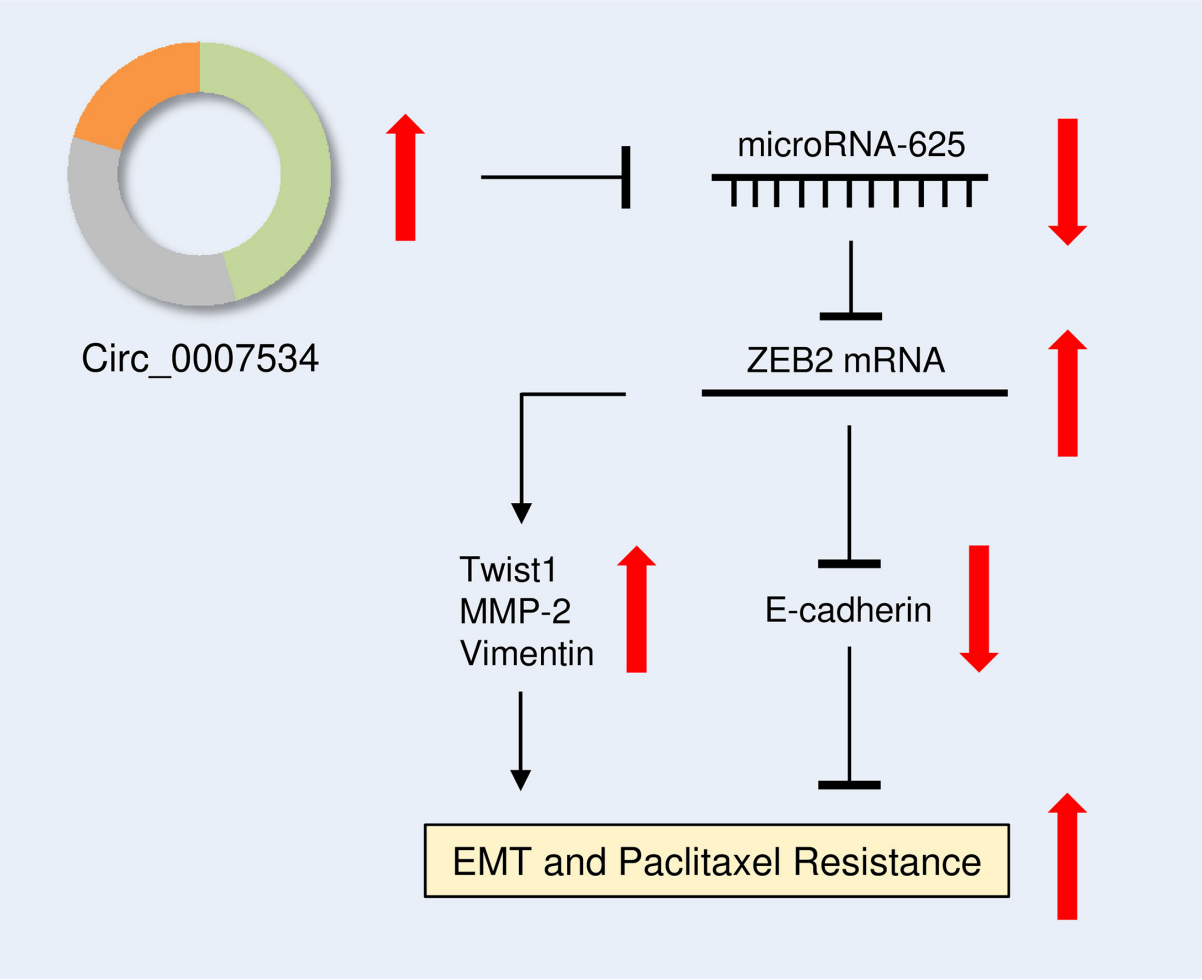
Diagram showing how circ_0007534 promotes EMT and chemoresistance in endometrial cancer *via* the miR-625/ZEB2 pathway.

The effect of ZEB2 expression on cervical cell invasion and EMT was also investigated. When ZEB2 levels were successfully decreased in HEC-1 cells after transfection with siRNA targeting ZEB2, the levels of *E-cadherin* were elevated, and the levels of *Twist1*, *Vimentin*, and *MMP2* were reduced (**Figure 9A**). The suppression of ZEB2 expression decreased HEC-1 cell invasion (**Figure 9B**). However, overexpression of ZEB2 repressed *E-cadherin* expression and upregulated the expression of *Twist1*, *Vimentin*, and *MMP2* in Ishikawa cells (**Figure 9C**). Additionally, ZEB2 overexpression promoted Ishikawa cell invasion (**Figure 9D**). Collectively, these findings suggested that oncogenic circ_0007534 facilitates EMT, invasion, and Paclitaxel resistance in endometrial cancer through the miR-625/ZEB2 pathway (**Figure 10**).

## Discussion

The failure of endometrial cancer treatment is closely associated with resistance to chemotherapy (such as Paclitaxel) (23). The mechanisms of Paclitaxel resistance are complex, including the aberrant expression of miRNAs and circRNAs (17, 24). However, the roles of miRNAs and circRNAs in endometrial cancer chemoresistance are largely unknown. The expression of circ_0007534 was found to be higher in diverse cancer tissues than in adjacent normal tissues (19–22). However, there have been no reports on the relationship between circ_0007534 and endometrial cancer chemoresistance or the role of circ_0007534 in endometrial cancer cells. The current study is the first to investigate the cellular roles of circ_0007534 in endometrial cancer progression and suggest an association between circ_0007534 overexpression and Paclitaxel resistance.

At present, the lack of effective biomarkers for diagnosis and prognosis, as well as therapeutic targets, are major issues in the prevention and treatment of endometrial cancer. CircRNAs have more stable structures than linear transcripts and are found in human blood, bodily fluids, and tissues (16–18). These circRNAs are expected to be promising diagnostic markers or therapeutic targets for human cancers (16–22). In this study, we discovered that circ_0007534 was overexpressed in endometrial cancer tissues and cell lines. More importantly, we found that a high circ_0007534 level was associated with more aggressive cancer phenotypes and a poor prognosis in endometrial cancer patients. According to our study, circ_0007534 has the potential to be a new biomarker for predicting invasion and metastasis and worse prognosis in endometrial cancer.

EMT has been identified as an important process in cancer metastasis and chemoresistance (4, 5). Additionally, chemotherapy resistance has been observed in cancer cells with EMT phenotypes and a greater ability to invade (25). Therefore, we focused on the effect of circ_0007534 on EMT induction and Paclitaxel resistance. Our *in vivo* and *in vitro* experiments showed that circ_0007534 promoted endometrial cancer cell proliferation, invasion, EMT, and Paclitaxel resistance. This is the first study that links circ_0007534 overexpression to endometrial cancer chemoresistance, possibly *via* the EMT process.

The roles of “miRNA sponges” have been widely investigated in recent years, and some circRNAs have been discovered to contain miRNA binding sites (26). Previous studies have reported that silencing circ_0009035 inhibits endometrial cancer cell proliferation, migration, invasion, and radioresistance *in vitro* while slowing tumor growth *in vivo* (27). Moreover, circ_0023404 enhances endometrial cancer metastasis and chemoresistance by regulating VEGF-A and autophagy signaling by sponging miR-5047 (28). The current study used bioinformatics analysis to determine that circ_0007534 might act as a sponge to influence miR-625 expression. This result was further confirmed by the luciferase reporter assay and real-time PCR assay. Furthermore, our functional assays showed that by acting as a sponge for miR-625, circ_0007534 promotes endometrial cancer cell proliferation, invasion, and Paclitaxel resistance. In line with our current results, a previous study has verified the direct binding between circ_0007534 and miR-625 in pancreatic cancer (22). By sponging miR-625, circ_0007534 greatly improved the ability of pancreatic cancer cells to proliferate, migrate, and invade, and it prevented cell apoptosis (22). Our findings support the oncogenic role of circ_0007534 in endometrial cancer tumorigenesis, progression, and chemoresistance. However, the roles and the underlying mechanisms by which circ_0007534 affects endometrial cancer are largely unknown.

Through post-transcriptional regulation of gene expression, miRNAs participate in a variety of cell activities during tumor development and chemoresistance (29). Lower miR-625 expression was found in endometrial cancer specimens compared with normal tissues (13). By targeting ALDH1A1, miR-625 has been shown to reverse multidrug resistance in gastric cancer cells (30). In addition, miR-625 inhibits the growth, migration, invasion, and EMT of lung cancer cells by targeting Resistin (14). In skin melanoma cells, miR-625 directly targeted YY1 to suppress cell proliferation, migration, invasion, and EMT (31). It has been shown that ZEB2 is a target of miR-625 in breast cancer (32). Consistent with these data, our results showed that overexpression of miR-625 in endometrial cancer cells suppressed EMT and Paclitaxel resistance, at least by downregulating the expression of ZEB2.

Existing research on *ZEB2* has shown that it serves as a novel oncogene to accelerate endometrial cancer progression (33, 34). Transfection of lung cancer and hepatocellular carcinoma cells with ZEB2 siRNA significantly decreased cancer cell viability in response to Paclitaxel treatment (9, 10). In this study, we wondered whether modulation of ZEB2 levels could have an impact on the EMT properties and the sensitivity of endometrial cancer cells to Paclitaxel. We have revealed that silencing of ZEB2 can effectively make endometrial cancer cells more sensitive to Paclitaxel treatment and lead to the downregulation of Twist1, MMP2, and vimentin and upregulation of E-cadherin. Therefore, decreasing ZEB2 levels might sensitize refractory endometrial cancer to Paclitaxel. Future research is needed to explore this possibility.

Multiple miRNAs (including miR-1270 and miR-377) can regulate the levels of ZEB2 in endometrial cancer (33, 34). Using the dual-luciferase reporter assay, we discovered that miR-625 was a key miRNA that directly binds to *ZEB2* mRNA and represses its expression in endometrial cancer cells. This study has shown a possible mechanism that accounts for the aberrant induction of ZEB2 expression in endometrial cancer patients. We speculated that the introduction of miR-625 might be a new treatment method for reversing Paclitaxel resistance in patients with endometrial cancer.

## Conclusion

Taken together, our results constructed a circ_0007534/miR-625/ZEB2 signaling, which drives endometrial cancer progression and chemoresistance by modulating several key EMT-related genes, including *Twist1*, *MMP2*, *E-cadherin*, and *Vimentin* (**Figure 10**). The results of this study imply that creating circ_0007534 detection and intervention strategies holds a promise for improving endometrial cancer diagnosis and treatment.

## Data availability statement

The original contributions presented here are included in the article. Further inquiries can be directed to the corresponding author.

## Ethics statement

The studies involving human participants or animals were reviewed and approved by the Ethics Committee of Second Affiliated Hospital of Nanchang University. The patients/participants provided their written informed consent to participate in this study. The animal study was reviewed and approved by the Animal Care and Use Committee of Second Affiliated Hospital of Nanchang University.

## Author contributions

SL designed the study. HY and YH performed the experiments. HY and SL wrote the manuscript. All authors contributed to the article and approved the submitted version.

## Funding

This work was supported by a grant from the Natural Science Foundation of Jiangxi Province, China (20202BABL206104).

## Acknowledgments

We thank TCGA for making the data publicly available.

## Conflict of interest

The authors declare that the research was conducted in the absence of any commercial or financial relationships that could be construed as a potential conflict of interest.

## Publisher’s note

All claims expressed in this article are solely those of the authors and do not necessarily represent those of their affiliated organizations, or those of the publisher, the editors and the reviewers. Any product that may be evaluated in this article, or claim that may be made by its manufacturer, is not guaranteed or endorsed by the publisher.
